# High-Temperature Tensile and Creep Behavior of Polypropylene/Poly(butylene terephthalate) Blends: Matrix Fibrillation and Interfacial Debonding

**DOI:** 10.3390/polym18182282

**Published:** 2026-09-18

**Authors:** Mio Kudo, Mai Ishikawa, Hirotaka Horiguchi, Shinya Goto, Hiromu Saito

**Affiliations:** 1 Materials Engineering R & D Division, DENSO CORPORATION, Kariya-shi 448-8661, Aichi, Japan; mio.tamura.j4c@jp.denso.com (M.K.);; 2 Department of Applied Chemistry, Tokyo University of Agriculture and Technology, Koganei-shi 184-8588, Tokyo, Japan; 3 Department of Industrial Technology and Innovation, Tokyo University of Agriculture and Technology, Koganei-shi 184-8588, Tokyo, Japan

**Keywords:** polymer blend, polypropylene, poly(butylene terephthalate), tensile property, creep resistance, matrix fibrillation, deformation mechanism, interfacial adhesion

## Abstract

We investigated the deformation and tensile behavior of crystallized polypropylene (PP)/poly(butylene terephthalate) (PBT) blends across a wide range of PBT compositions. Low-PBT-content blends (100/1 and 100/3 PP/PBT) exhibited enhanced yield stresses at room temperature, as well as superior or comparable tensile ductility and creep resistance at 100 °C compared to neat PP. These improvements were driven by robust interfacial adhesion established via crystallization-induced mechanical interlocking derived from surface-induced nucleation of PP from small PBT domains. This robust interfacial adhesion, suggested by a high-temperature shift in the αc-relaxation reflecting enhanced interfacial constraint in dynamic mechanical analysis, promoted a specific deformation sequence wherein matrix fibrillation preceded interfacial debonding, thereby suppressing transverse craze propagation and enabling stable large-strain drawing. Conversely, the high-PBT-content blend (100/20 PP/PBT) exhibited brittle fracture behavior governed by a weakest-link-dominated failure mechanism, in which premature interfacial debonding at coarse domain boundaries under intense triaxial stress concentration triggered extensive transverse crazing prior to matrix fibrillation. These findings provide valuable fundamental insights for designing tough, heat-resistant polymer blends without conventional chemical compatibilizers, which can serve as a conceptual framework for upcycling mixed polymer waste streams.

## 1. Introduction

Polymer blending is widely recognized as one of the most efficient and economically viable strategies for developing advanced materials by tailoring property balances [1,2]. Furthermore, because diverse polymer species inevitably become mixed during disposal and collection—particularly in the dismantling and shredding stream of end-of-life vehicles (ELVs)—polymer blending serves as a practical approach for reusing waste plastics via mechanical recycling [3,4,5]. However, because most polymer pairs are thermodynamically immiscible, they inherently exhibit coarse phase-separated morphologies and weak interfacial adhesion [6,7]. This weak interface severely hinders stress transfer across phases, often causing premature failure and compromising their overall mechanical performance [7,8,9,10].

During the deformation of polymer blends, local stress concentration plays a decisive role in dictating fracture behavior. Dispersed domains in the matrix constrain the lateral Poisson contraction of the surrounding matrix during stretching due to stiffness mismatch. This constraint generates transverse tensile stresses alongside the primary tensile stress, creating an intense triaxial stress state around the domains [11,12,13,14,15,16]. Such triaxial stress concentration restricts matrix shear deformation, thereby promoting microvoid nucleation, craze transformation, and interfacial debonding. The unhindered growth and coalescence of these microvoids and debonded interfaces ultimately lead to catastrophic brittle failure [11,14,16,17,18]. Consequently, controlling and reinforcing the interfacial structure has become a central objective in polymer blend research.

To overcome these performance limitations, adding reactive or block copolymer compatibilizers has been widely employed to refine phase morphology and enhance interfacial adhesion [3,5,6,19,20,21,22,23,24,25,26]. While these conventional approaches successfully improve macroscopic tensile properties and impact strength, they frequently introduce processing complexities, increase material costs, and in some cases, interfere with the intrinsic crystallization behavior of the constituent polymers [6,27,28]. Although the relationship between interfacial adhesion and mechanical properties has been extensively investigated [29,30,31], and strong interfacial adhesion is recognized to suppress premature interfacial debonding, it remains fundamentally unclear how the interfacial state dictates the competition between interfacial debonding and energy-dissipating matrix deformation (e.g., matrix fibrillation [14,32,33,34,35]) under large drawing and sustained loading.

Isotactic polypropylene (PP) represents an ideal matrix polymer as a widely utilized thermoplastic crystalline polymer owing to its low density, excellent processability, and cost-effectiveness [36,37]. Indeed, PP accounts for over 40% of the plastics used in automotive interior and exterior components. Meanwhile, poly(butylene terephthalate) (PBT), a high-performance engineering plastic, is widely employed for electrical connectors and housings in vehicle components [6]. Although PP and PBT are rarely combined within a single engineered component, they may become blended in mixed waste streams during end-of-life vehicle (ELV) disposal and material recycling processes [6]. While poly(ethylene terephthalate) (PET) is more ubiquitous in municipal packaging waste, PBT represents a far more relevant aromatic polyester in the context of automotive engineering plastics and ELV recycling streams. Furthermore, compared to PET, PBT possesses a lower melting temperature and a much faster crystallization rate, enabling co-extrusion without inducing severe thermal degradation of the PP matrix. Therefore, the PP/PBT system serves as an ideal and practically relevant model for investigation. Because PP and PBT are completely immiscible and exhibit distinctly different crystallization and deformation characteristics [6,7], the PP/PBT system also serves as an ideal model for investigating interfacial mechanics in immiscible blends. In this regard, it has been reported that PP crystallizes from the surface of electrospun PBT fibers [38], forming a transcrystalline layer that enhances interfacial adhesion [39,40,41,42]. Recently, we discovered that PBT domains induce surface-induced nucleation of PP, leading to significant spherulitic refinement and direct epitaxial growth of PP crystals from PBT domain surfaces, which markedly accelerated PP crystallization [43]. The resulting enhanced interfacial adhesion provided low-PBT-content blends (100/1 and 100/3 PP/PBT) with higher yield stresses at room temperature compared to neat PP [43]. While that previous study established the physical mechanism of crystallization and the resulting crystalline morphology, the macroscopic mechanical durability and microscopic deformation/fracture mechanisms governed by this crystallization-induced interface remain entirely unexplored, particularly under large strains, elevated temperatures, and prolonged creep loading. Crucially, whether the strong interfacial adhesion achieved through surface-induced nucleation can withstand severe high-temperature drawing and sustained stress without conventional chemical compatibilizers represents an essential question for practical engineering applications.

To resolve these fundamental issues, the present study significantly advances beyond our previous morphological work by systematically investigating the high-temperature (100 °C) tensile drawing and creep behaviors across a wide range of PBT compositions, complemented by dynamic mechanical analysis (DMA), optical and polarized optical microscopy, scanning electron microscopy (SEM), and differential scanning calorimetry (DSC). The obtained results were discussed in terms of the competitive micromechanical sequence between interfacial debonding and local matrix deformation. Ultimately, this work using model PP/PBT blends provides vital mechanistic insights, establishing a fundamental guideline toward the future design of tough, heat-resistant recycled polymer materials through crystallization-induced interfacial engineering without conventional chemical compatibilizers.

## 2. Materials and Methods

### 2.1. Materials

Isotactic polypropylene (PP) is a high-strength grade of polypropylene formulated for automotive components that was purchased from Sumitomo Chemical Co., Ltd. (Tokyo, Japan) (MFR = 35 g/10 min at 230 °C under a 21.6 N), containing commercial stabilizers to suppress thermal and oxidative degradation during melt processing. Poly(butylene terephthalate) (PBT) was purchased from Polyplastics Co., Ltd. (Tokyo, Japan) (MFR = 12–20 g/10 min at 260 °C under a 21.6 N).

Prior to melt-mixing, the PBT pellets were dried under vacuum at 100 °C for 5 h to remove residual moisture and prevent hydrolytic degradation. The PP and PBT were melt-mixed at a kneading temperature of 280 °C using a twin-screw extruder (Process 11, Thermo Fisher Scientific Inc., Waltham, MA, USA; screw diameter *D* = 11 mm, *L*/*D* = 40) at a screw speed of 300 rpm and a feed rate of 1.0 kg/h. The barrel temperature profile from hopper to die was 160/280/280/280/280/280/280 °C. The extruded strands were cooled and pelletized. The blend compositions of PP/PBT blends prepared in this study were 100/1, 100/3, 100/5, 100/10, and 100/20 (*w*/*w*). For optical and polarized optical microscopy observations, film specimens with a thickness of approximately 0.2 mm were prepared by compression-molding at 250 °C for 10 min under vacuum using a hot-press machine (11FD, Imoto Machinery Co., Ltd., Kyoto, Japan), followed by isothermal melt crystallization at 130 °C for 20 min and subsequent rapid quenching in a water bath.

### 2.2. Methods

#### 2.2.1. Optical and Polarized Optical Microscopy

The morphological features of the film specimens were observed using an optical microscope (BX53, Olympus Corporation, Tokyo, Japan) equipped with a CCD camera (DP74, Olympus Corporation, Tokyo, Japan). A high numerical aperture (NA) objective lens (Plan-Apochromat 50×, NA = 0.95, Olympus Corporation, Tokyo, Japan) was used to achieve high spatial resolution and a narrow depth of field, enabling the observation of fine structures. Observations were performed under unpolarized light without a crossed polarizer, as well as under polarized light using crossed polarizers equipped with a sensitive tint plate (optical path difference of 530 nm).

#### 2.2.2. Tensile Tests

Dumbbell-shaped film specimens were prepared for tensile testing using a die-cutter according to ASTM D 1708 with a gauge length of 12 mm and a gauge width of 5.0 mm. Specimen thickness was measured at three locations within the gauge section using a digital micrometer, yielding an average thickness of approximately 0.2 mm. Tensile tests were conducted at 23 °C using an Instron-type tensile testing machine (Strograph VES05D, Toyo Seiki Seisaku-sho, Ltd., Tokyo, Japan) at a crosshead speed of 50 mm/min. Additionally, tensile tests were conducted at 100 °C using a thermal stretching apparatus equipped with a tensile load cell and a sensor interface (Taiatsu Techno Corporation, Tokyo, Japan) at a crosshead speed of 50 mm/min. Manual screw grips with flat clamping faces lined with #600 sandpaper were utilized to prevent specimen slippage. For tests at 100 °C, the specimens were allowed to thermally equilibrate inside the chamber for 5 min before loading. At least three specimens were tested for each sample condition. Tensile properties were evaluated in terms of engineering stress and engineering strain, where engineering strain was determined from the crosshead displacement divided by the initial gauge length, and engineering stress was calculated using the initial cross-sectional area. The compliance of the testing machine was confirmed to be negligible owing to the small applied tensile loads relative to the load cell capacity, and engineering values were adopted to provide a standardized basis for comparing the non-uniform drawing and cavitation behaviors across all blends up to large strains (>400–600%).

#### 2.2.3. DMA Measurement

Dynamic mechanical analysis (DMA) was carried out using a dynamic mechanical analyzer (DMA 1, Mettler-Toledo International Inc., Columbus, OH, USA) in the tensile mode. Rectangular specimens measuring 5 mm in width, 0.2 mm thickness, and 30 mm in length were evaluated with an initial gap distance of 5 mm. Measurements were performed from −40 to 155 °C at a heating rate of 2 °C/min under a constant oscillatory frequency of 1 Hz. The storage modulus *E*′ was obtained as a function of temperature.

#### 2.2.4. SEM Observation

The deformed structures of the blend films were observed using a scanning electron microscope (SEM; Ethos NX5000, Hitachi High-Tech Corp., Tokyo, Japan). Prior to observation, the film specimens were sectioned into ultra-thin slices using an ultramicrotome (Ultracut-UCT, Leica Biosystems Nussloch GmbH, Nussloch, Germany) and stained with RuO_4_ vapor under vacuum at 20 °C. SEM was operated at an accelerating voltage of 3 kV under vacuum.

#### 2.2.5. DSC Measurement

Differential scanning calorimetry (DSC) measurements were performed on specimens at a heating rate of 10 °C/min in a nitrogen atmosphere using a DSC Q200 (TA Instruments, New Castle, DE, USA). The mass of each test specimens was about 3.5 mg.

#### 2.2.6. Creep Tests

Creep tests were performed at a constant temperature of 100 °C in an oven (DVS403, Yamato Scientific Co., Ltd., Tokyo, Japan) under a constant nominal engineering tensile stress of 7.5 MPa. Dumbbell-shaped specimens identical to those used in the tensile tests were employed. The time-dependent elongation was monitored using a displacement gauge (DP-500G, Tokyo Measuring Instruments Laboratory Co., Ltd., Tokyo, Japan; a calibration coefficient of 0.0500 mm/1 × 10^−6^) connected to a PC-controlled dynamic strain meter (DC-004P, Tokyo Measuring Instruments Laboratory Co., Ltd., Tokyo, Japan). The nominal tensile stress of 7.5 MPa was imposed by applying a total tensile force of approximately 7.5 N across the initial gauge cross-sectional area of the specimen (1.0 mm^2^). The total applied force of 7.5 N accounted for a dead weight of 610 g (approximately 6.0 N) and the tension of 1.5 N from the displacement gauge. Prior to loading, each specimen was thermally equilibrated at 100 °C for 5 min. Immediately after fracture, each specimen was retrieved and rapidly quenched in water. Creep measurements were performed at least three times for each condition.

## 3. Results and Discussion

### 3.1. Room Temperature Tensile Behavior

**Figure 1:**
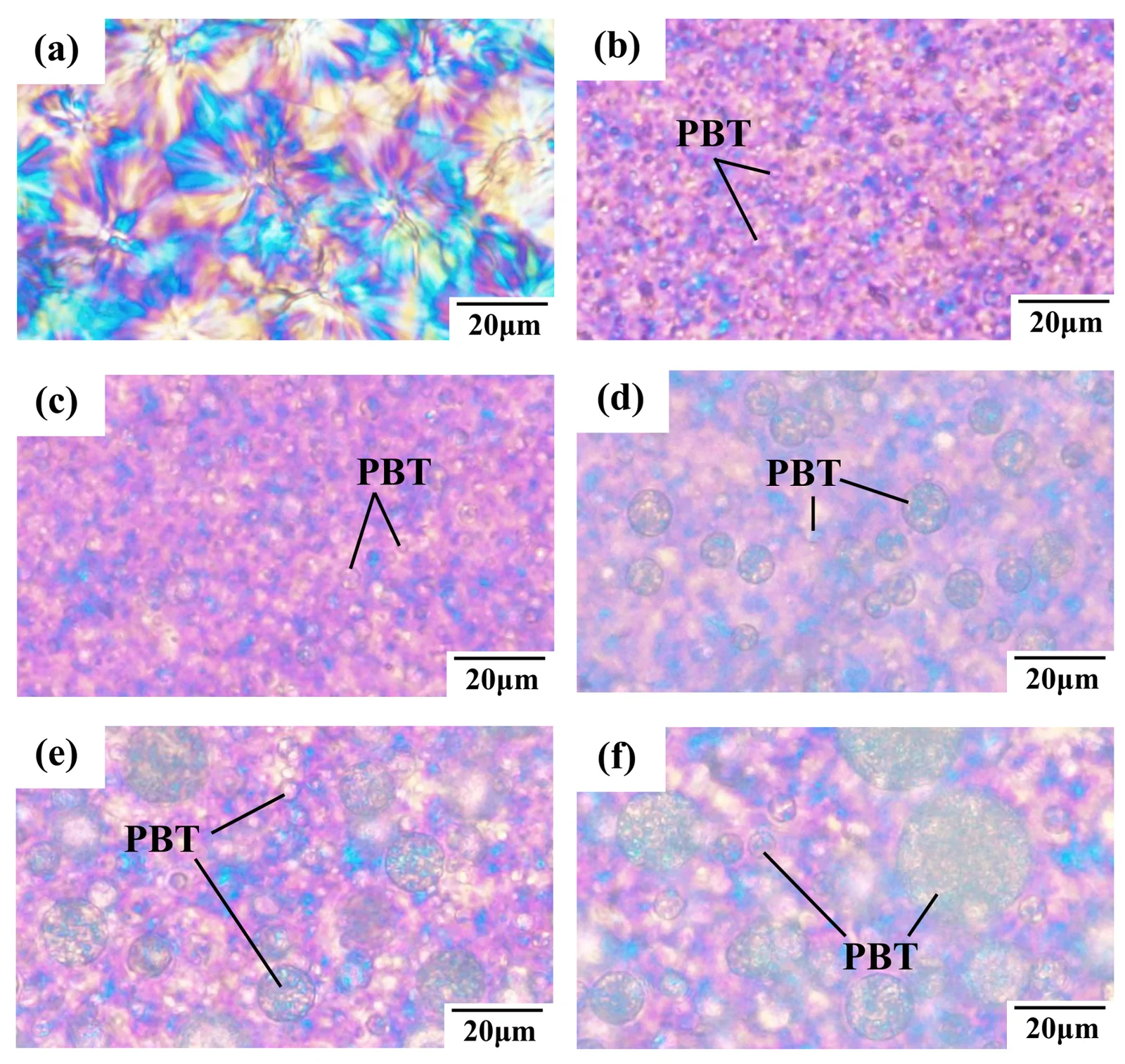
Polarized optical micrographs of PP/PBT blends with various compositions crystallized at 130 °C after melting at 250 °C: (**a**) 100/0 (neat PP), (**b**) 100/1, (**c**) 100/3, (**d**) 100/5, (**e**) 100/10, and (**f**) 100/20.

Figure 1 shows polarized optical micrographs of the polypropylene (PP)/poly(butylene terephthalate) (PBT) blends obtained after melt crystallization at 130 °C following melting at 250 °C. The PBT domain diameters and inter-domain ligament distances (expressed as mean ± standard deviation, SD), quantitatively evaluated from the uncropped micrographs of Figure 1, are summarized in Table 1. Neat PP exhibited coarse spherulites with diameters ranging from 20 to 30 μm, displaying distinct blue and yellow interference colors (Figure 1a). In the 100/1, 100/3, and 100/5 PP/PBT blends, small spherical PBT domains with distinct interference colors and diameters ranging from 1 to 7 µm were dispersed within the PP matrix, which showed pale interference colors (Figure 1b–d). The mean PBT domain diameter increased with increasing PBT content—yielding 1.0, 2.8, and 6.4 μm for the 100/1, 100/3 and 100/5 blends, respectively. The size of the PP spherulites was markedly smaller than that in neat PP, resulting in poorly defined spherulite boundaries. As demonstrated in our previous studies, this spherulite refinement is attributed to the nucleating agent effect of PBT via surface-induced crystallization, with small PBT domains located at the centers of the PP spherulites [43].

In contrast, the 100/10 and 100/20 PP/PBT blends exhibited coarse PBT domains ranging from 10 to 30 µm, coexisting with smaller domains of around 3 µm (Figure 1e,f). The mean diameters of the coarse PBT domains increased with increasing PBT content—yielding 15.4 and 17.9 μm for the 100/10 and 100/20 blends, respectively—accompanied by a progressive reduction in the inter-domain ligament distance (Table 1). Due to the presence of these coexisting small PBT domains, the blends exhibited accelerated crystallization via a nucleating agent effect, as shown in Figure S1 (Supplementary Materials), suggesting surface-induced nucleation of PP from the small PBT domains [43]. Consequently, the PP spherulites in these blends were markedly smaller than those in neat PP. Notably, the coarse PBT domains were larger than the average diameter of the surrounding PP spherulites. Based on these two-dimensional morphological observations, it is hypothesized that these coarse PBT domains predominantly reside outside or between the PP spherulites, rather than being completely encapsulated within individual spherulite centers as observed in the low-PBT-content blends.

**Figure 2:**
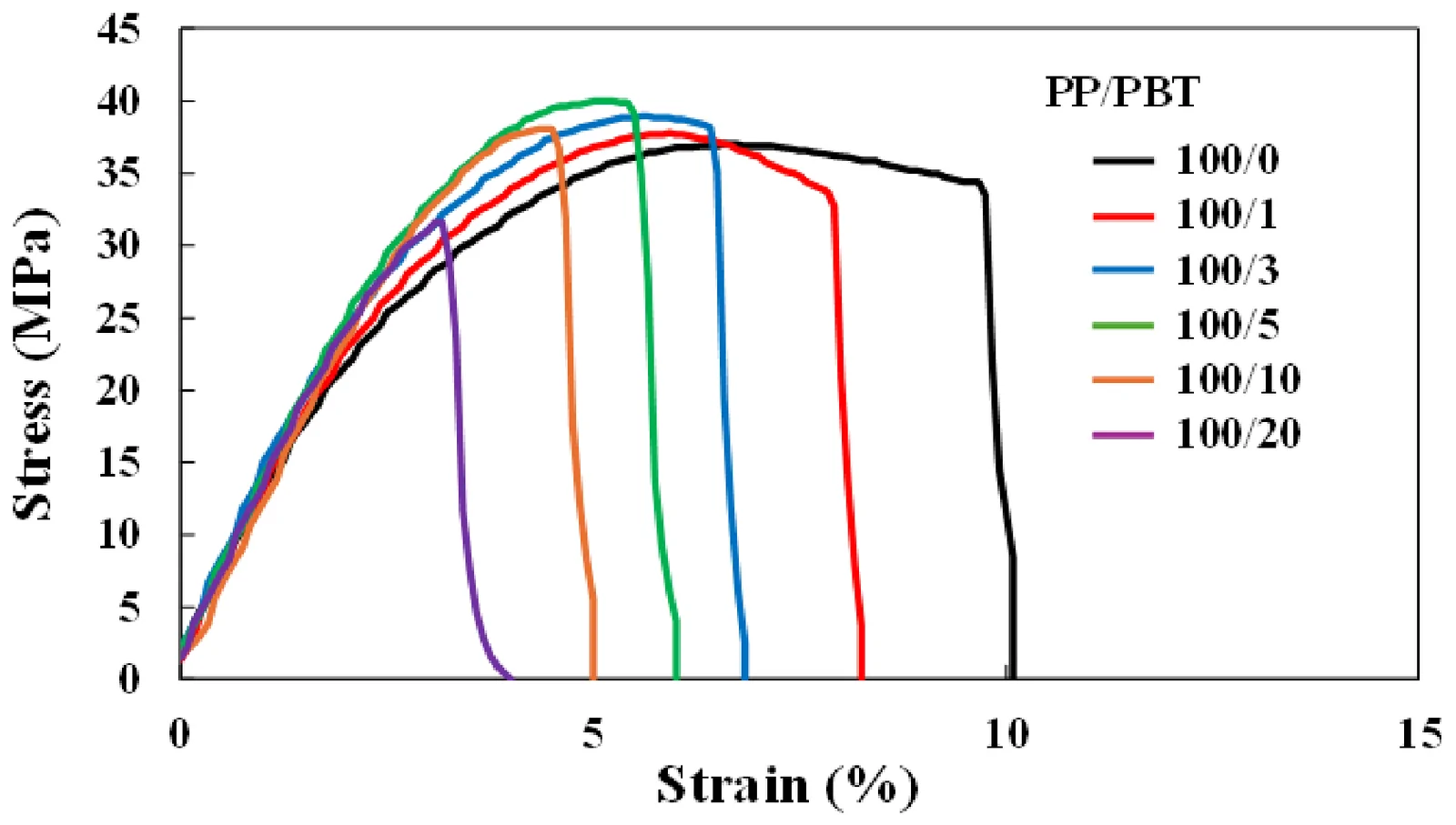
Stress–strain curves of PP/PBT blends with various compositions measured at room temperature.

Figure 2 shows representative stress–strain curves of the crystallized PP/PBT blends with different compositions measured at room temperature. The key tensile properties and their statistical distributions (mean ± standard deviation, SD), including yield stress, yield strain, and elongation at break, are comprehensively summarized in Table 2. For neat PP, the stress increased linearly with strain up to approximately 2% strain. Beyond this linear region, the rate of stress increase diminished, exhibiting a non-linear stress increase up to the yield point at around 7% strain, before decreasing and culminating in fracture at a strain of approximately 10%. It should be noted that the PP used in this study is a high-strength grade designed for specific automotive components, and its properties differ from those of the more ductile PP commonly evaluated in conventional studies [44]. All PP/PBT blends also exhibited a linear elastic response up to 2% strain, followed by non-linear deformation. Beyond 2% strain, the reduction in stress increase in the low-PBT-content blends (100/1, 100/3, and 100/5) was significantly less pronounced than in neat PP. The sustained load-bearing capacity in the non-linear region up to the yield point depends on effective stress transfer across the interface. The robust interfacial adhesion resulting from surface-induced nucleation prevents early micro-debonding, thereby enabling the low-PBT-content blends to achieve higher yield stresses than neat PP without adding conventional chemical compatibilizers. Conversely, the high-PBT-content blends (100/10 and 100/20) underwent catastrophic fracture prior to or near the yielding point, resulting in a lower elongation at break than neat PP; specifically, the elongation at break was reduced to 5.5% and 4.1% for the 100/10 and 100/20 PP/PBT blends, respectively (compared to 8.3% for neat PP).

**Figure 3:**
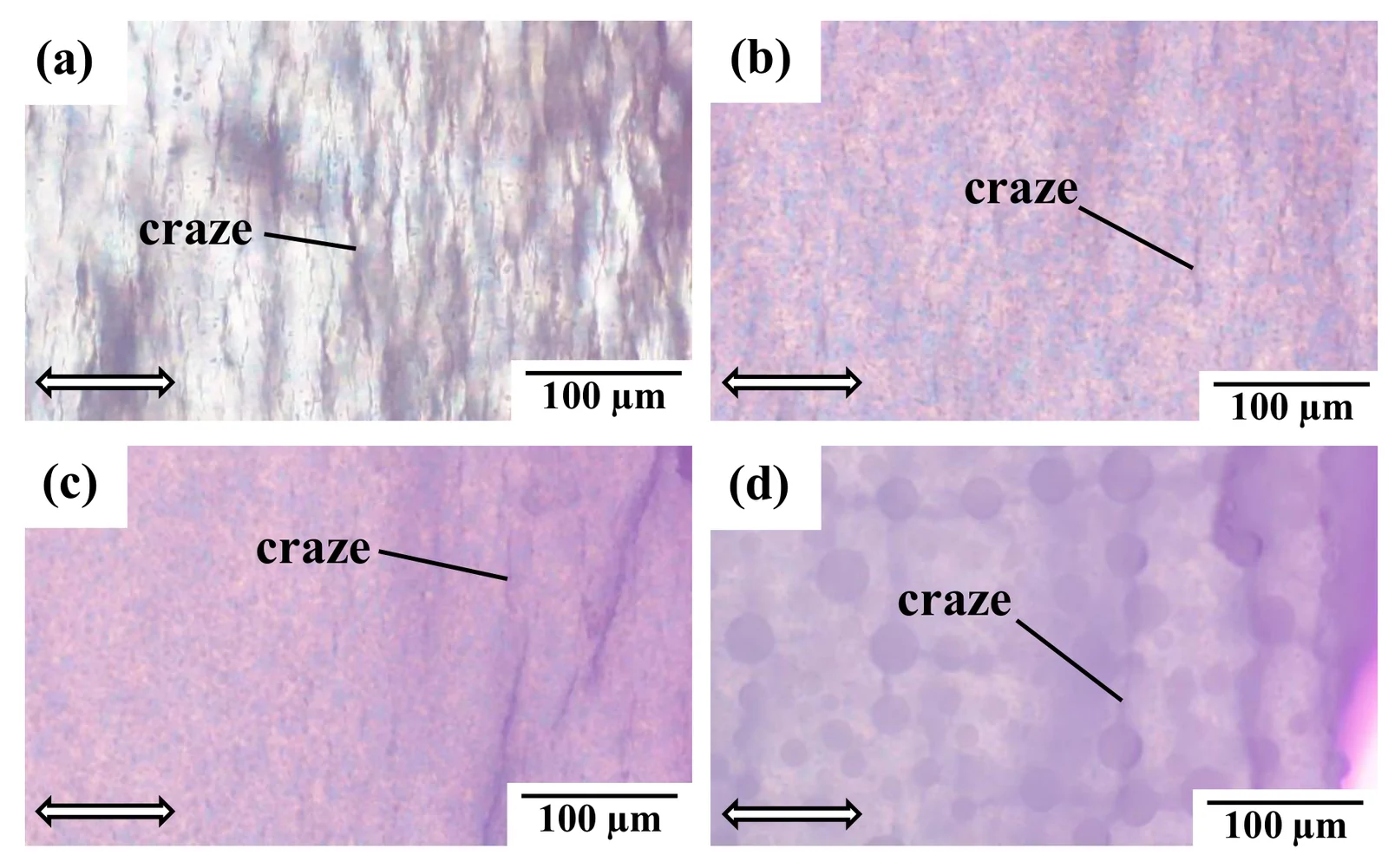
Polarized optical micrographs of PP/PBT blends with various compositions taken near the fracture surfaces after tensile testing at room temperature: (**a**) 100/0, (**b**) 100/1, (**c**) 100/3, and (**d**) 100/20. The arrow indicates the tensile direction.

Figure 3 shows polarized optical micrographs of neat PP and the PP/PBT blends after tensile testing, corresponding to the deformation in Figure 2. Here, the 100/1 and 100/3 blends, along with the 100/20 blend, are presented as representative low- and high-PBT-content blends, respectively. Observations were conducted near the fracture surfaces of the specimens. Upon deformation, neat PP developed numerous large crazes aligned perpendicular to the tensile direction (Figure 3a). The low elongation at break (~10% strain) shown in Figure 2 is caused by this localized initiation and propagation of large crazes. Consistent with the initial morphology shown in Figure 1b,c, the size of PBT domains within the deformed regions tended to increase with higher PBT content. In the 100/1 and 100/3 PP/PBT blends, only fine, localized crazes were observed (Figure 3b,c), and both their size and number density were significantly reduced compared to neat PP, indicating that craze formation was effectively suppressed. Furthermore, no interfacial debonding was detected in these low-PBT-content blends. This suppression of interfacial debonding is attributed to the robust adhesion between the PP matrix and small PBT domains promoted by surface-induced nucleation [43]. These observations suggest that, in low-PBT-content blends, strong interfacial adhesion suppresses craze initiation at low strains, thereby enhancing yield stress.

In contrast, the high-PBT-content blend (100/20 PP/PBT), which contained coarse PBT domains, exhibited pronounced crazes within the PP matrix regions bridging adjacent coarse PBT domains (Figure 3d). While the overall number of crazes was smaller than in neat PP, the size of individual crazes was considerably larger. Many of the crazes were aligned perpendicular to the tensile direction and interconnected neighboring coarse PBT domains, indicating that craze formation was induced by transverse tensile stress arising from the triaxial stress concentration, which was further amplified by premature interfacial debonding at the coarse PBT domain surfaces. Although the filler-like reinforcing effect of the PBT domains initially mitigated stress reduction at low strains, this premature interfacial debonding due to presence of coarse PBT domains induced catastrophic craze propagation, leading to brittle fracture prior or near the yielding point. Consequently, the elongation at break was reduced from 5.5% for the 100/10 PP/PBT to 4.1% for the 100/20 PP/PBT, corresponding to the presence of coarser PBT domains and a shortened inter-domain ligament distance (Table 1).

**Figure 4:**
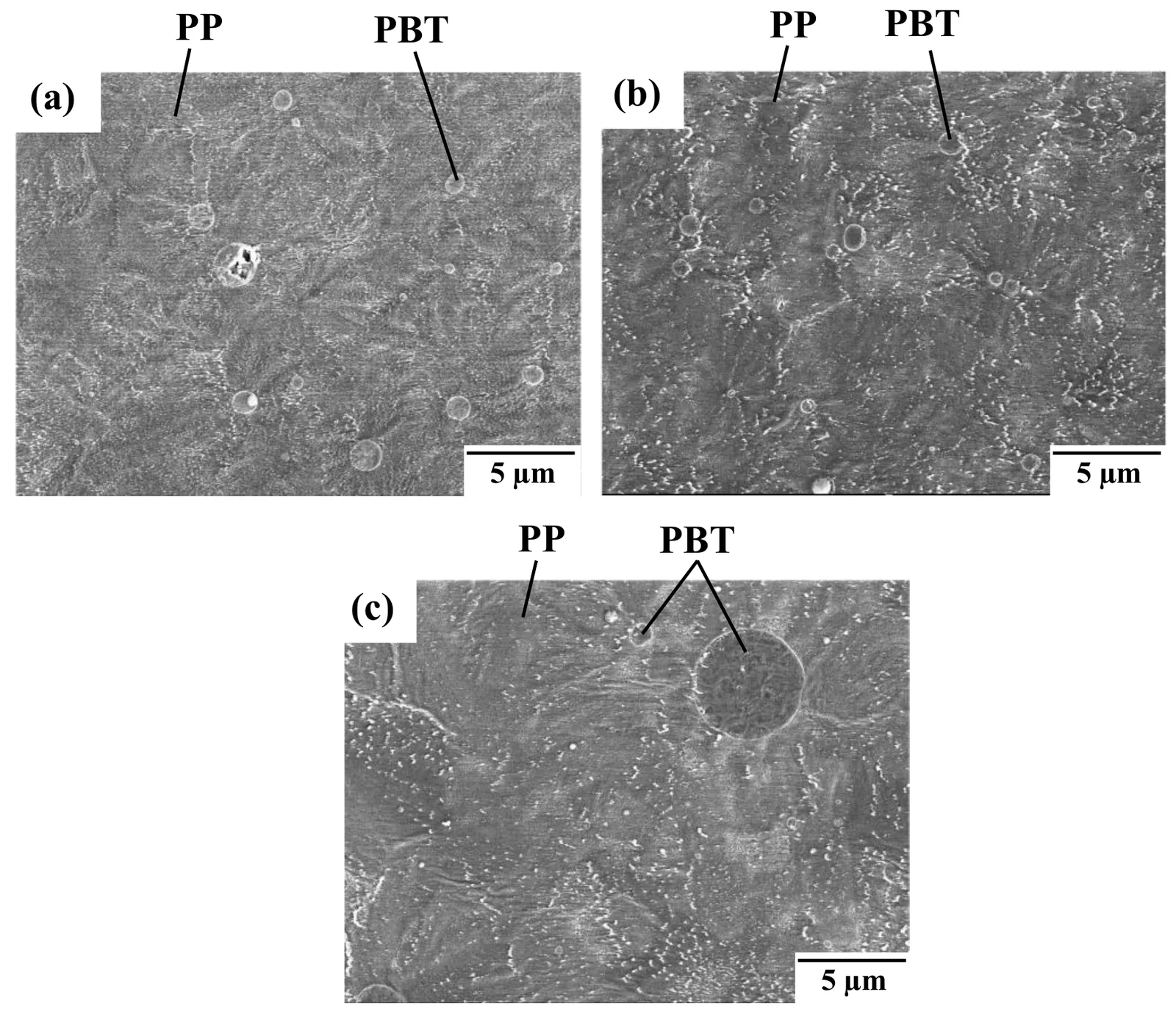
SEM micrographs of PP/PBT blends with various compositions taken near the fracture surfaces after tensile testing at room temperature: (**a**) 100/1, (**b**) 100/3, and (**c**) 100/20. The tensile direction is aligned horizontally along the equator, corresponding to Figure 3.

Figure 4 presents SEM micrographs taken near the fracture surfaces of the PP/PBT blends after tensile testing at room temperature. In the low-PBT-content blends (100/1 and 100/3 PP/PBT), no void formation or crazes initiating from the surfaces of the PBT domains were observed, suggesting intact interfacial adhesion. In contrast, for the high-PBT-content blend (100/20 PP/PBT), pronounced crazing was visible in the PP matrix near the interface of the coarse PBT domains, extending both parallel and perpendicular to the tensile axis due to triaxial stress concentration. These fractographic features suggest that premature interfacial debonding at coarse PBT domains served as primary stress concentration sites that induced catastrophic failure. It should be noted that the bright particulate features observed in the SEM images, which were much smaller than the PBT domains, are presumed to originate from surface irregularities introduced during specimen preparation, as they were detected in secondary electron images. Furthermore, comparison with backscattered electron images revealed no evidence of penetration of staining agents into these regions, confirming that these features are not crazes.

Since neat PP exhibits brittle behavior at room temperature, discussing the mechanisms of interfacial debonding solely based on room temperature deformation is insufficient. Therefore, to further elucidate the deformation and fracture mechanisms, the stress–strain behavior was subsequently investigated at elevated temperature above the onset temperature of αc-relaxation, where molecular chain mobility within the crystalline region of PP is activated.

### 3.2. High Temperature Tensile Behavior

**Figure 5:**
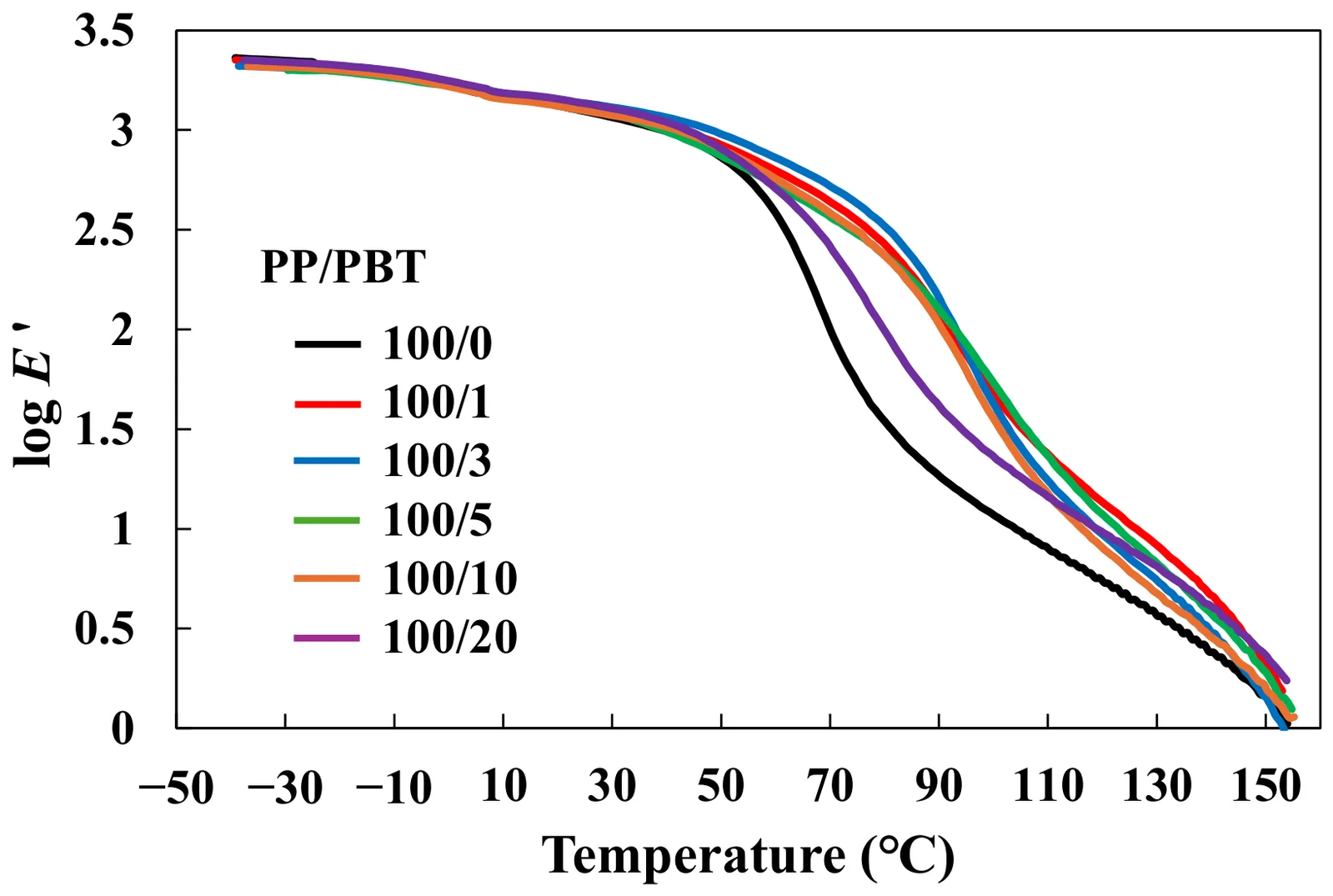
Temperature dependence of the storage modulus (*E*′) of PP/PBT blends with various compositions.

Figure 5 shows the temperature dependence of the storage modulus (*E*′) for the PP/PBT blends with different compositions obtained by dynamic mechanical analysis (DMA). Here, the curves were vertically shifted and superimposed at −40 °C to facilitate a clear comparison of the temperature-dependent reduction in *E*’. In the low-temperature region (−30 to 50 °C), no significant differences in *E*′ were observed among the samples. For neat PP, *E*’ exhibited a steep decline starting at around 65 °C, corresponding to the onset of the αc-relaxation associated with the activation of molecular chain mobility within the PP crystalline region [44,45,46,47,48]. Notably, this temperature-dependent drop in *E*’ shifted markedly toward higher temperatures in the blends; specifically, the αc-relaxation temperatures estimated from the onset of the *E*’ drop for neat PP, 100/1, 100/3, 100/5, 100/10, and 100/20 PP/PBT were 55 °C, 71 °C, 74 °C, 70 °C, 71 °C, and 57 °C, respectively. All DMA measurements were conducted at least in duplicate for each composition to confirm experimental reproducibility. It should be noted that while loss tangent (tan *δ*) peaks are commonly employed for sharp transitions such as the glass transition, the αc-relaxation of PP spans a very broad temperature range, resulting in a diffuse, low-intensity tan *δ* shoulder at elevated temperatures where baseline fluctuations hinder precise peak determination. Therefore, evaluating αc-relaxation temperature from the onset of the *E*’ drop, as schematically illustrated in Figure S2 (Supplementary Materials), which provides direct benchmark for the start of macroscopic mechanical softening in the crystalline matrix.

Previous studies have established that the αc-relaxation of a crystalline polymer matrix shifts to higher temperatures when strong interfacial adhesion suppresses the mobility of polymer chains near the interface in polymer blends [49] and polymer composites [50,51,52,53]. Although other structural factors such as lamellar thickness and crystallinity can potentially influence the αc-relaxation temperature, the observed shift cannot be attributed to lamellar thickening. As revealed by the DSC thermograms of the undrawn specimens (Section 3.2, Figure 9), neat PP exhibited a sharp melting peak, whereas the blends displayed a broadened endotherm with a low-temperature foot, reflecting a larger population of thinner lamellae formed via accelerated surface-induced nucleation. Since thinner lamellae possess a lower activation barrier for crystalline chain mobility, morphological effects alone would typically shift the αc-relaxation toward lower temperatures. Therefore, this high-temperature shift particularly pronounced in the low-PBT-content blends (100/1, 100/3 and 100/5 PP/PBT) cannot be explained by lamellar variations, but is consistent with interfacial constraint, suggesting that the molecular mobility of PP crystalline chains near the domain boundaries is effectively restricted by the strong interfacial adhesion established by surface-induced nucleation [43]. Due to the intrinsic thermodynamic immiscibility and the lack of reactive functional groups, chemical bonding is absent between the two phases. Instead, the enhanced interfacial adhesion in this blend is considered to be primarily governed by crystallization-induced mechanical interlocking, as demonstrated in polymer composites [54,55,56] and blend [57]. During melt crystallization, PP lamellar crystals nucleate directly on the surfaces of PBT domains via surface-induced nucleation and grow epitaxially into the surrounding matrix. These densely grown PP lamellae intimately penetrate and conform to the surface irregularities of the PBT domains, acting as physical anchors. This geometric interlocking of crystalline lamellae at the phase boundary mechanically restricts interfacial sliding and resists debonding under applied stress, thereby establishing robust interfacial integrity even without chemical compatibilizers.

Conversely, the shift was smaller in the high-PBT-content blend (100/20 PP/PBT), indicating a weaker constraining effect due to poorer interfacial adhesion at the surfaces of coarse domains, despite the presence of smaller PBT domains that induce surface nucleation. The presence of coarse PBT domains generate severe local triaxial stress concentrations that exceed the local interfacial load-bearing limit, initiating early micro-debonding and catastrophic craze propagation before the surrounding ductile matrix can undergo strain-induced fibrillation. Therefore, the brittle behavior of the high-PBT-content blend should not be simply classified as an absence of interfacial adhesion, but rather as weakest-link-dominated failure induced by the coarse domain. Furthermore, no shift in the αc-relaxation has been reported in PP/PBT blends with higher PBT contents (60/40–40/60 PP/PBT) [23], suggesting the absence of significant interfacial adhesion.

**Figure 6:**
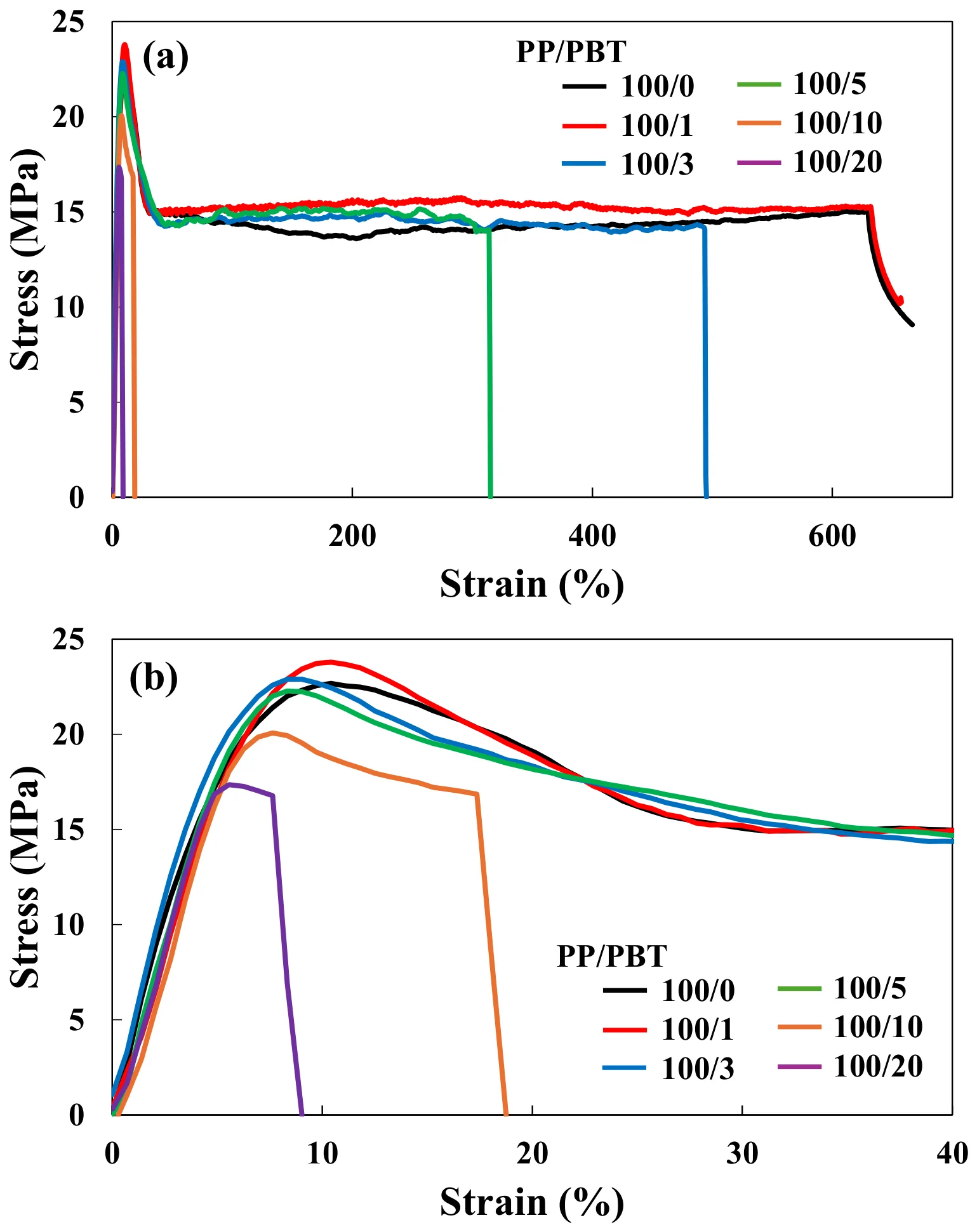
Stress–strain curves of PP/PBT blends with various compositions measured at 100 °C: (**a**) full strain range of 0–700% and (**b**) corresponding enlarged strain of 0–40%.

Figure 6a presents representative stress–strain curves of the PP/PBT blends measured at 100 °C. The key tensile properties and their statistical distributions (mean ± standard deviation, SD), including yield stress, yield strain, and elongation at break, are comprehensively summarized in Table 3. This temperature is above the onset temperature of αc-relaxation, where the mobility of PP crystalline chains is thermally activated, allowing for the evaluation of the interfacial mechanical integrity under large deformation. Owing to enhanced crystalline chain mobility, the brittle response of neat PP at room temperature transitioned into ductile deformation at 100 °C. The low-PBT-content blends (100/1, 100/3, and 100/5 PP/PBT) similarly exhibited ductile behavior, undergoing stable drawing up to high strains, although molecular mobility was lower than neat PP due to the higher αc-relaxation temperature (Figure 5). This demonstrates that the strong interfacial constraint suggested by DMA effectively prevents premature micro-debonding without the addition of conventional chemical compatibilizers, thereby ensuring that the PP/PBT interface does not act as a fracture initiation site even under severe deformation, as discussed later. In contrast, the high-PBT-content blends (100/10 and 100/20 PP/PBT) exhibited severe embrittlement and underwent catastrophic fracture prior to or near the yield point even at 100 °C, leading to a dramatic reduction in elongation at break compared to neat PP; specifically, the elongation at break was reduced to 10.0% and 7.9% for the 100/10 and 100/20 PP/PBT blends, respectively (compared with >600% for neat PP).

Figure 6b shows an enlarged view of the stress–strain curves in the initial strain range of 0–40%, corresponding to Figure 6a. The low-PBT-content blends maintained yield stresses comparable to or slightly higher than that of neat PP. Conversely, the high-PBT-content blend fractured prematurely before reaching the yield point. This stark difference indicates that the interface in the high-PBT-content blend fails catastrophically under large macroscopic deformations despite the PP matrix being ductile.

**Figure 7:**
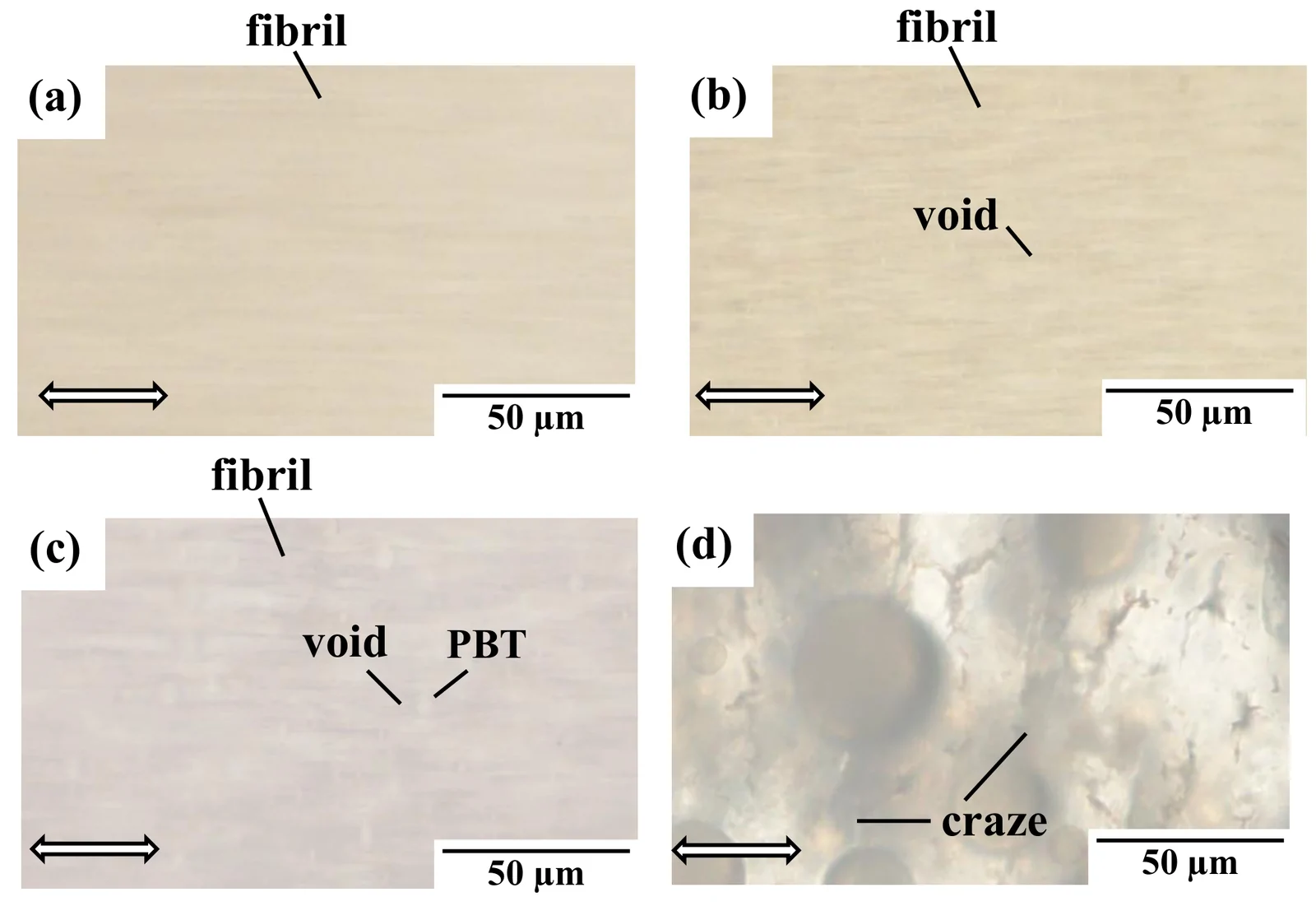
Optical micrographs of PP/PBT blends with various compositions taken near the fracture surfaces after tensile testing at 100 °C: (**a**) 100/0 (neat PP), (**b**) 100/1, (**c**) 100/3, and (**d**) 100/20. The arrow indicates the drawing direction. Cor-responding polarized optical micrographs are shown in Figure S2 (Supplementary Materials).

To clarify the microscopic deformation mechanisms at high temperature, the deformed morphologies were characterized. Figure 7 shows optical micrographs taken near the fracture surface of neat PP and the PP/PBT blends after tensile testing at 100 °C. Here, the 100/1 and 100/3 blends, along with the 100/20 blend, are presented as representative low- and high-PBT-content blends, respectively. Neat PP developed highly oriented fibrillar structures aligned along the drawing axis (Figure 7a), which were formed during post-yield plastic deformation as typically observed in neat crystalline polymers [58,59]. Crazes aligned perpendicular to the drawing direction were entirely absent, indicating that strain-induced matrix fibrillation effectively dissipated local stress concentrations and facilitated high ductility with an elongation at break exceeding 500%. Similarly, the low-PBT-content blends displayed fibrillar structures (Figure 7b,c). Although a small number of isolated microvoids were observed in the 100/3 PP/PBT blend (Figure 7c), they did not induce premature brittle fracture. These results suggest that catastrophic fracture propagation initiated by microvoids is effectively suppressed by strain-induced matrix fibrillation.

In contrast, no fibrillar structures were observed in the high-PBT-content blend; instead, large crazes were formed (Figure 7d). These macro-crazes bridged adjacent coarse PBT domains perpendicular to the drawing direction, similar to the brittle fracture pattern observed at room temperature (Figure 3d). This catastrophic fracture behavior can be attributed to two synergistic factors: domain size and inter-domain distance. The coarse PBT domains with diameters of 10–30 μm not only create severe local stress concentrations but also shorten the inter-domain matrix ligament distance (Table 1). When initial debonding occurs at the interfaces of these coarse PBT domains, the overlapping stress fields between closely spaced adjacent domains generate intense triaxial stress in the intervening PP matrix [11,12,13,14,15,16,17,18]. The local stress concentration perpendicular to the drawing direction induced by this intense triaxial stress severely restricts plastic flow even at 100 °C (above onset temperature of αc-relaxation) and forces the PP matrix to undergo brittle craze growth rather than ductile fibrillation, resulting in catastrophic brittle fracture. Consequently, the elongation at break was reduced from 14.9% for the 100/10 PP/PBT to 8.8% for the 100/20 PP/PBT, corresponding to the presence of coarser PBT domains and a shortened inter-domain ligament distance (Table 1).

**Figure 8:**
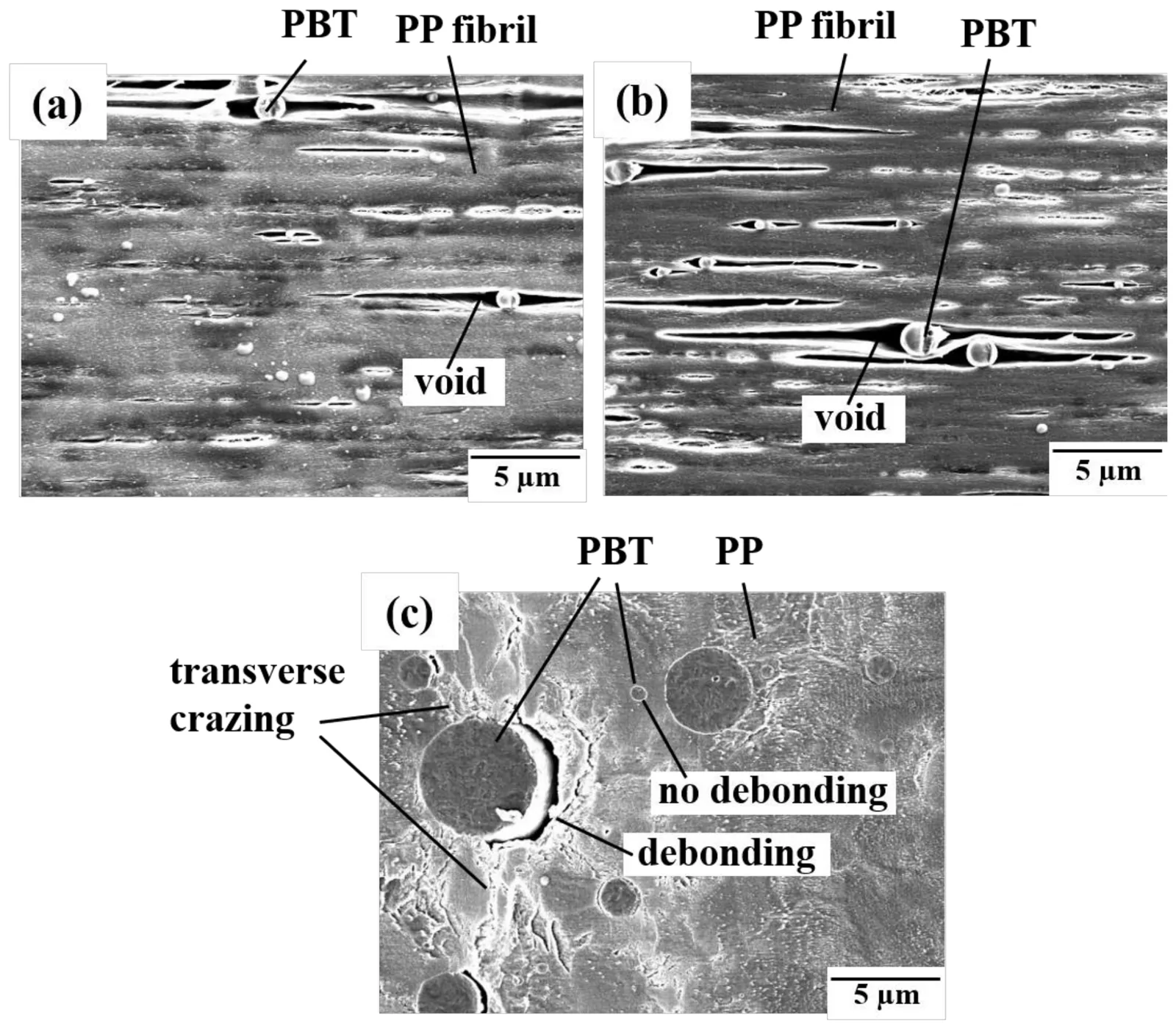
SEM micrographs of PP/PBT blends with various compositions taken near the fracture surfaces after tensile testing at 100 °C: (**a**) 100/1, (**b**) 100/3, and (**c**) 100/20. The drawing direction is aligned horizontally along the equator, corresponding to Figure 7.

Figure 8 presents SEM micrographs taken near the fracture surfaces of the PP/PBT blends after tensile testing at 100 °C. In the low-PBT-content blends (100/1 and 100/3 PP/PBT), longitudinal voids aligned along the drawing direction were observed, within which fine distorted PBT domains of sub-micrometer size were embedded (Figure 8a,b). This morphology is similar to that observed in deformed PP/ethylene–propylene–block copolymer blends [33], high density polyethylene/calcium carbonate blends [34], and PP/polyamide 6/polyethylene-octene elastomer blends [35], which is characteristic of matrix fibrillation [14,32]. These PBT domains exhibited rough, distorted surface rather than smooth, featureless pull-out surface, indicating that localized interfacial adhesion persisted during extensive plastic deformation. Dense fibrillar structures aligned along the drawing axis were clearly visible. Although longitudinal voids were generated by interfacial debonding, no crazes aligned perpendicular to the tensile direction were formed due to dominant strain-induced matrix fibrillation. These observations demonstrate that matrix fibrillation preceded extensive interfacial debonding, thereby enhancing resistance to tensile deformation and suppressing craze formation perpendicular to the drawing direction. Consequently, even when localized interfacial debonding was initiated during drawing, the tensile load was sustained up to high strains exceeding 400%.

In contrast, in the high-PBT-content blend (100/20 PP/PBT), coarse PBT domains debonded prematurely at the interface, initiating large crazes that propagated in various directions into the PP matrix (Figure 8c). These macro-crazes interconnected adjacent coarse PBT domains perpendicular to the tensile direction due to the intense triaxial stress concentration in the intervening PP matrix, as described previously. Conversely, some small PBT domains exhibited neither debonding nor induced transverse crazing owing to robust interfacial adhesion promoted by crystallization-induced mechanical interlocking derived from surface-induced nucleation. Neither fibrillar structures nor elongated longitudinal voids were observed, indicating that crazing occurred prior to the onset of strain-induced matrix fibrillation. Premature debonding at the surfaces of coarse PBT domains acted as sharp defects under triaxial stress concentration, thereby inducing craze growth and leading to brittle fracture governed by a weakest-link-dominated failure mechanism.

**Figure 9:**
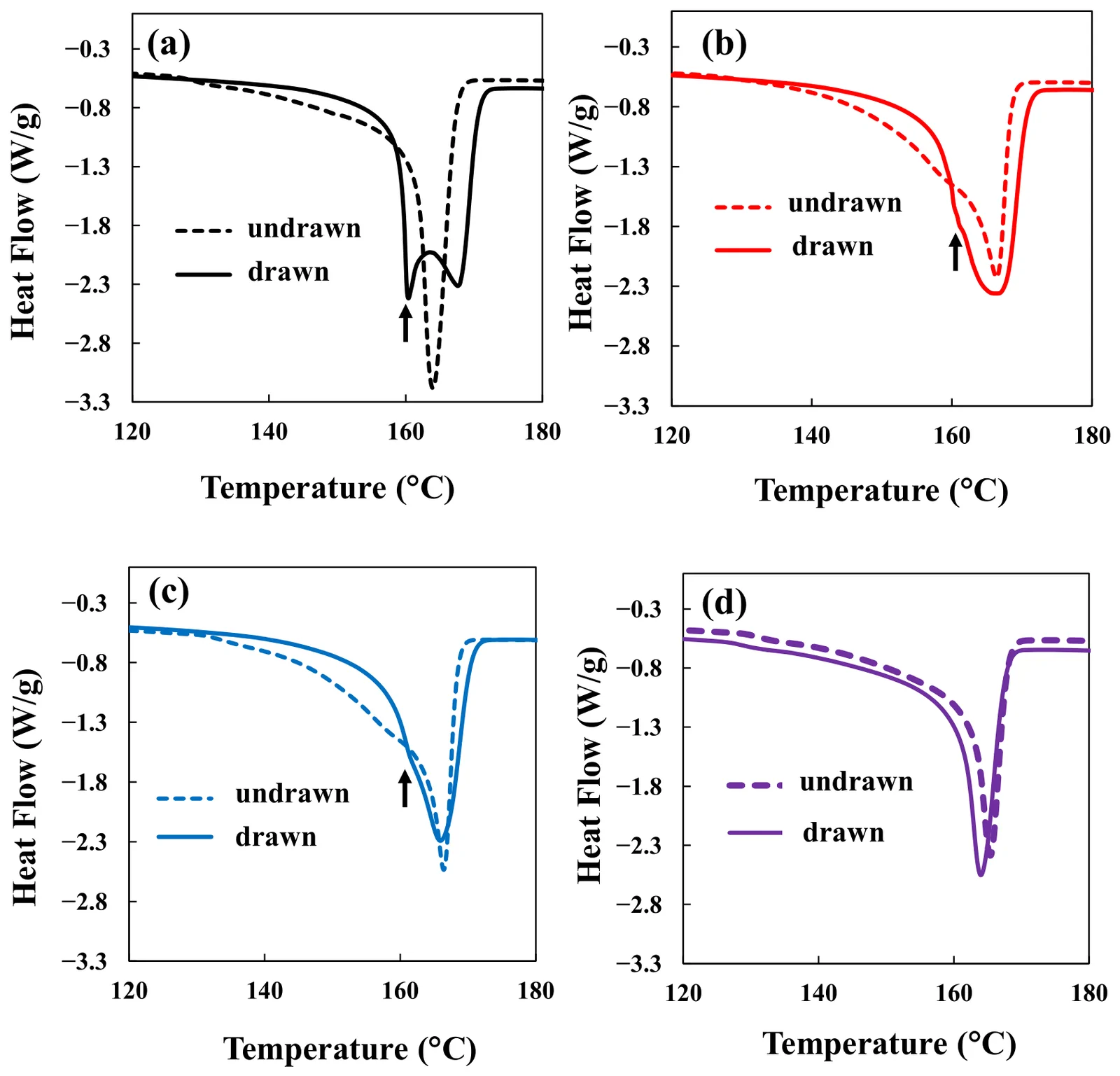
DSC thermograms of undrawn and drawn PP/PBT blends with various compositions, corresponding to Figure 7 and Figure 8: (**a**) 100/0 (neat PP), (**b**) 100/1, (**c**) 100/3, and (**d**) 100/20. The arrow indicates a peak attributed to fibrillation.

Strain-induced matrix fibrillation was further confirmed by DSC thermograms of the deformed specimens. For undrawn neat PP, a single melting peak was observed, which split into two distinct melting peaks upon drawing to 200% strain (Figure 9a). The lower- and higher-temperature melting peaks around 160 °C and 170 °C are assigned to thinner fibrillar crystallites formed during drawing and pre-existing thicker lamellae, respectively [44,60]. Additionally, drawing caused a reduction in the low-temperature foot of the melting peak due to the rearrangement of thin crystalline lamellae during fibrillation. Undrawn low-PBT-content blends (100/1 and 100/3 PP/PBT) initially exhibited a single melting peak with a broad low-temperature foot. Upon drawing up to 200% strain, a distinct shoulder peak appeared at around 160 °C, accompanied by a reduction in the broad low-temperature foot, while the main peak at around 170 °C remained almost unchanged (Figure 9b,c). The emergence of this shoulder peak provides supportive thermodynamic evidence of strain-induced matrix fibrillation, which complements the direct SEM and optical microscopic observations shown in Figure 7 and Figure 8. The intensity of this shoulder peak decreased with increasing PBT content, indicating that the dispersed PBT domains interfered to some extent with the uniform plastic drawing of the PP matrix. Although DSC alone cannot resolve the real-time temporal sequence of deformation, the presence of this distinct endothermic feature in the drawn low-PBT blends—contrasted with its absence in the 100/20 blend—is consistent with the morphological finding that energy-dissipating plastic deformation of the PP matrix was activated prior to premature macroscopic failure.

In contrast, no significant changes were observed in the melting thermograms of the high-PBT-content blend (100/20 PP/PBT) drawn up to its fracture strain (Figure 9d), suggesting the absence of strain-induced matrix fibrillation. This thermodynamic evidence supports that the high-PBT-content blend failed via premature debonding-induced crazing before matrix fibrillation could be activated, leading to catastrophic fracture prior to macroscopic yielding.

**Figure 10:**
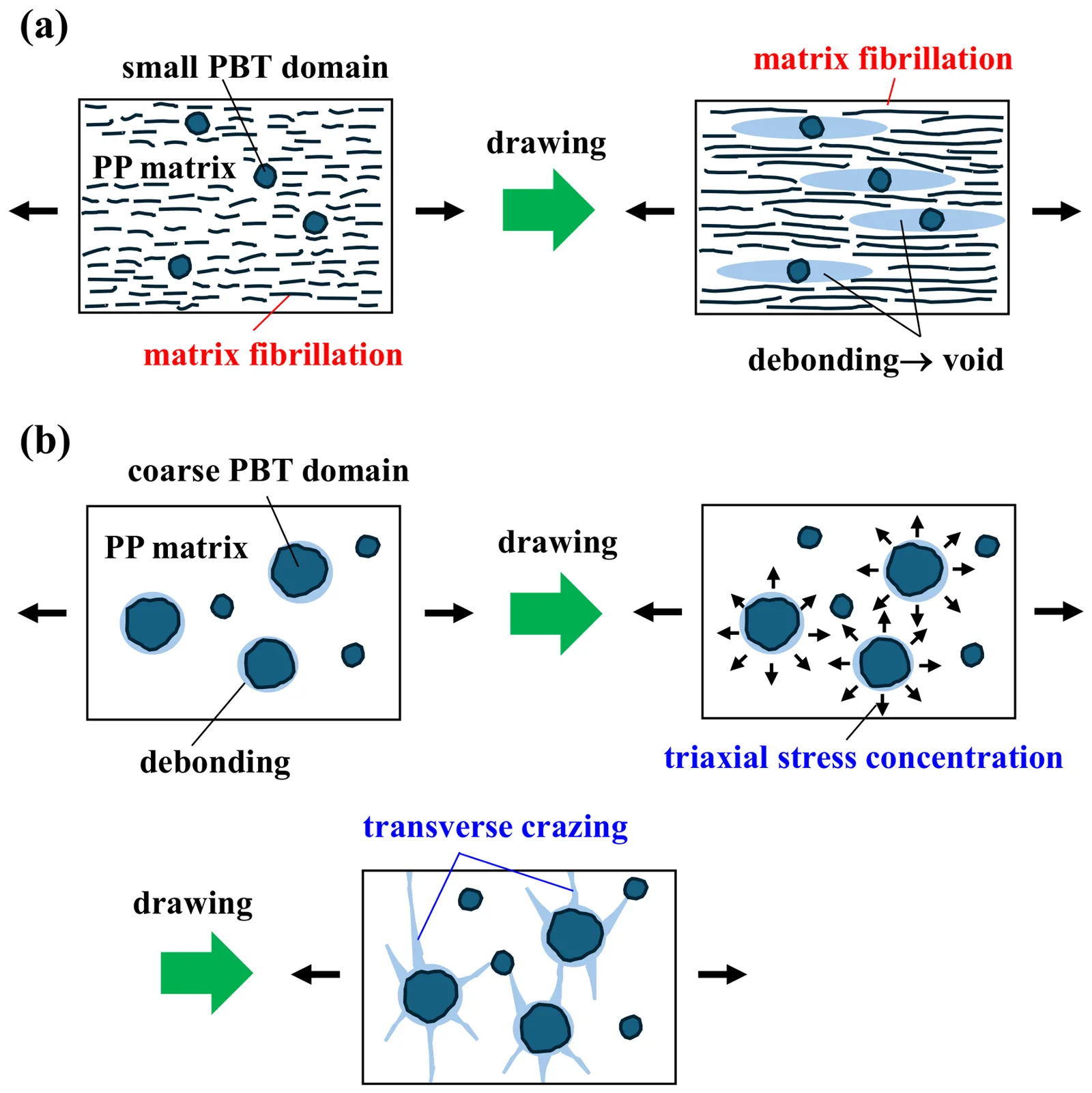
Schematic illustration of the high-temperature deformation and fracture mechanisms of PP/PBT blends: (**a**) strain-induced fibrillation prior to interfacial debonding and void formation (100/1 blend) and (**b**) transverse crazing caused by triaxial stress concentration (100/20 blend). The arrows indicate the tensile direction.

Thus, the low-PBT-content blends exhibited ductile behavior due to strain-induced matrix fibrillation preceded interfacial debonding and void formation, which was enabled by the robust interfacial adhesion achieved by crystallization-induced mechanical interlocking derived from surface-induced nucleation of PP crystallites from small PBT domains. Conversely, the high-PBT-content blend exhibited brittle behavior governed by a weakest-link-dominated failure mechanism, in which premature interfacial debonding around coarse PBT domains generated triaxial stress concentration that triggered transverse crazing prior to strain-induced matrix fibrillation. These contrasting deformation sequences are schematically illustrated in the structural models in Figure 10.

### 3.3. High Temperature Creep Behavior

**Figure 11:**
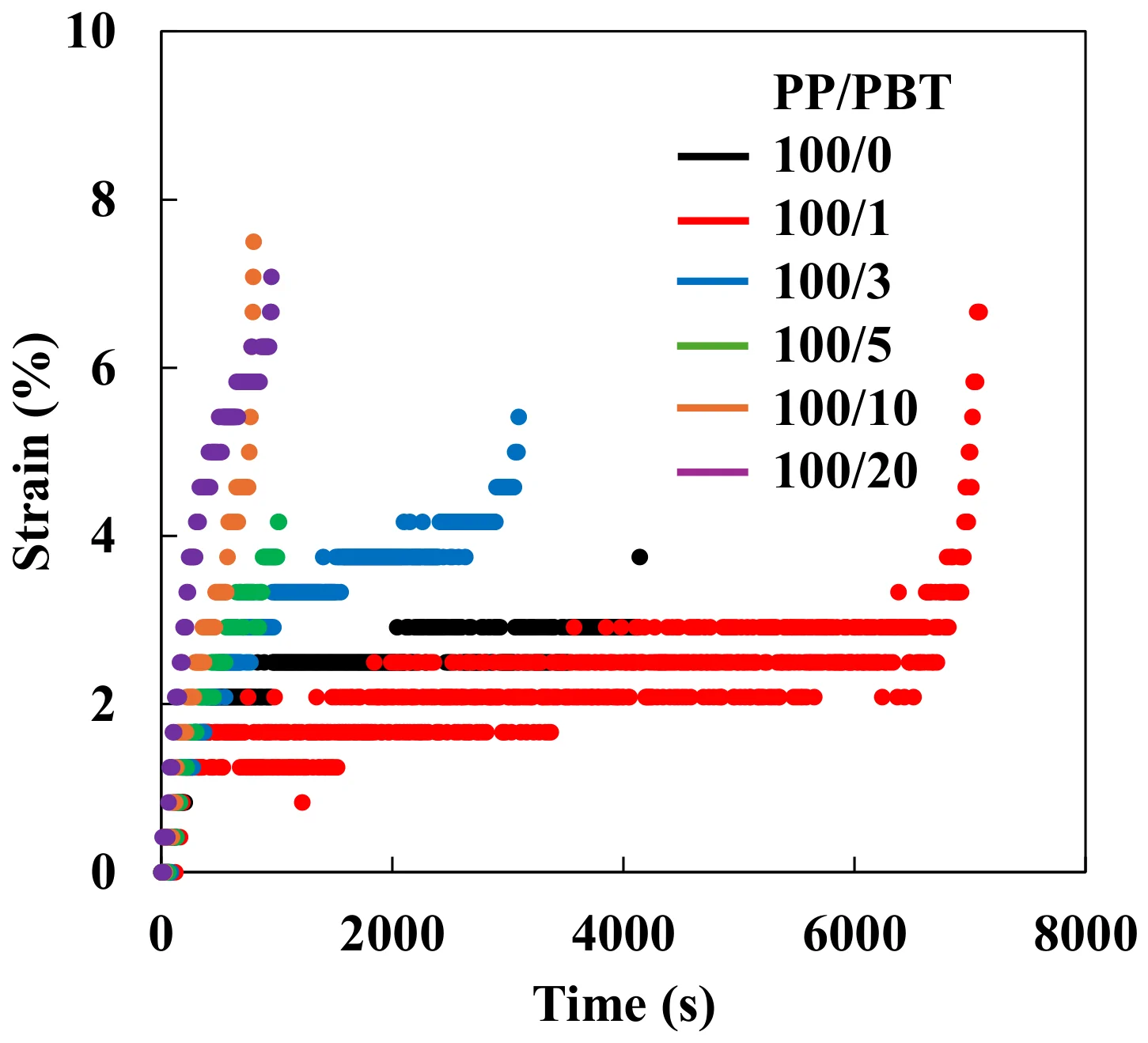
Time-dependent creep strain curves of PP/PBT blends with various compositions measured at 100 °C.

To further clarify the time-dependent deformation mechanisms and mechanical integrity under sustained loading at high temperature, creep tests under a constant tensile stress of 7.5 MPa were performed at 100 °C on the PP/PBT blends with different compositions. Here, the tensile stress of 7.5 MPa was approximately one-third of the yield stress of neat PP, as evaluated in Figure 6. The resulting time-dependent creep strain curves are shown in Figure 11. Due to the low magnitude of creep strain, despite testing above the onset temperature of αc-relaxation, and the finite strain resolution of the measurement system, the data points do not form a smooth, continuous curve; instead, they appear as a series of horizontally elongated step-like clusters that gradually shift toward longer times and higher strains. Despite this discrete data resolution, the overall profile clearly exhibits the characteristic three-stage creep behavior: an initial steep increase in strain (primary creep, Stage I), followed by a prolonged region of low, steady-state strain rate (secondary creep, Stage II), and finally an accelerated increase in strain leading to failure (tertiary creep, Stage III) [61,62,63,64].

The steady-state creep rate and creep rupture lifetime obtained at 100 °C under a constant tensile stress of 7.5 MPa are quantitatively summarized in Table 4. The steady-state creep rate was determined via linear regression of the strain-time curves within the minimum-slope interval in Stage II. Linear regression across the step-like clusters minimized the influence of instrument displacement resolution. The steady-state creep rate of the 100/1 PP/PBT blend was lower than that of neat PP, i.e., the steady-state creep rate for neat PP and the 100/1 PP/PBT blends were 3.6 × 10^−6^ s^−1^ and 2.0 × 10^−6^ s^−1^, respectively. Furthermore, the steady-state creep region of the 100/1 PP/PBT blend was noticeably longer than that of neat PP, thereby extending its creep rupture time, without the addition of conventional chemical compatibilizers. Specifically, the average fracture times obtained from three tests for neat PP and the 100/1 PP/PBT blends were 3376 s and 6491 s, respectively. This substantial enhancement in creep lifetime indicates superior creep resistance in the 100/1 blend. Meanwhile, the 100/3 PP/PBT blend exhibited a moderately higher steady-state creep rate (8.3 × 10^−6^ s^−1^) and a shorter Stage II region compared to neat PP (3.6 × 10^−6^ s^−1^). Consequently, the 100/3 blend achieved a slightly shorter average time-to-fracture of 2607 s (compared to 3376 s for neat PP).

With increasing PBT content beyond the 100/3 blend, the steady-state creep rate increased, while the creep rupture lifetime decreased. The 100/5 PP/PBT blend exhibited an accelerated creep rate compared to neat PP—its steady-state rate was increased to 18 × 10^−6^ s^−1^ (compared to 3.6 × 10^−6^ s^−1^ for neat PP)—despite exhibiting lower crystalline chain mobility inferred from its higher αc-relaxation temperature (αc-relaxation temperature of neat PP and 100/5 blend were 55 °C and 70 °C, respectively). The high-PBT-content blend (100/20 PP/PBT) exhibited a markedly accelerated creep rate than neat PP from the early stage of deformation—its steady-state rate was increased to 42 × 10^−6^ s^−1^ (compared to 3.6 × 10^−6^ s^−1^ for neat PP). Although it eventually progressed to Stage III and displayed a larger apparent fracture strain—attributable to the extensive opening of macro-crazes as described later—its average time-to-fracture was reduced to 867 s (compared to 3376 s for neat PP). This accelerated creep deformation indicates the poor creep resistance of the high-PBT-content blend.

**Figure 12:**
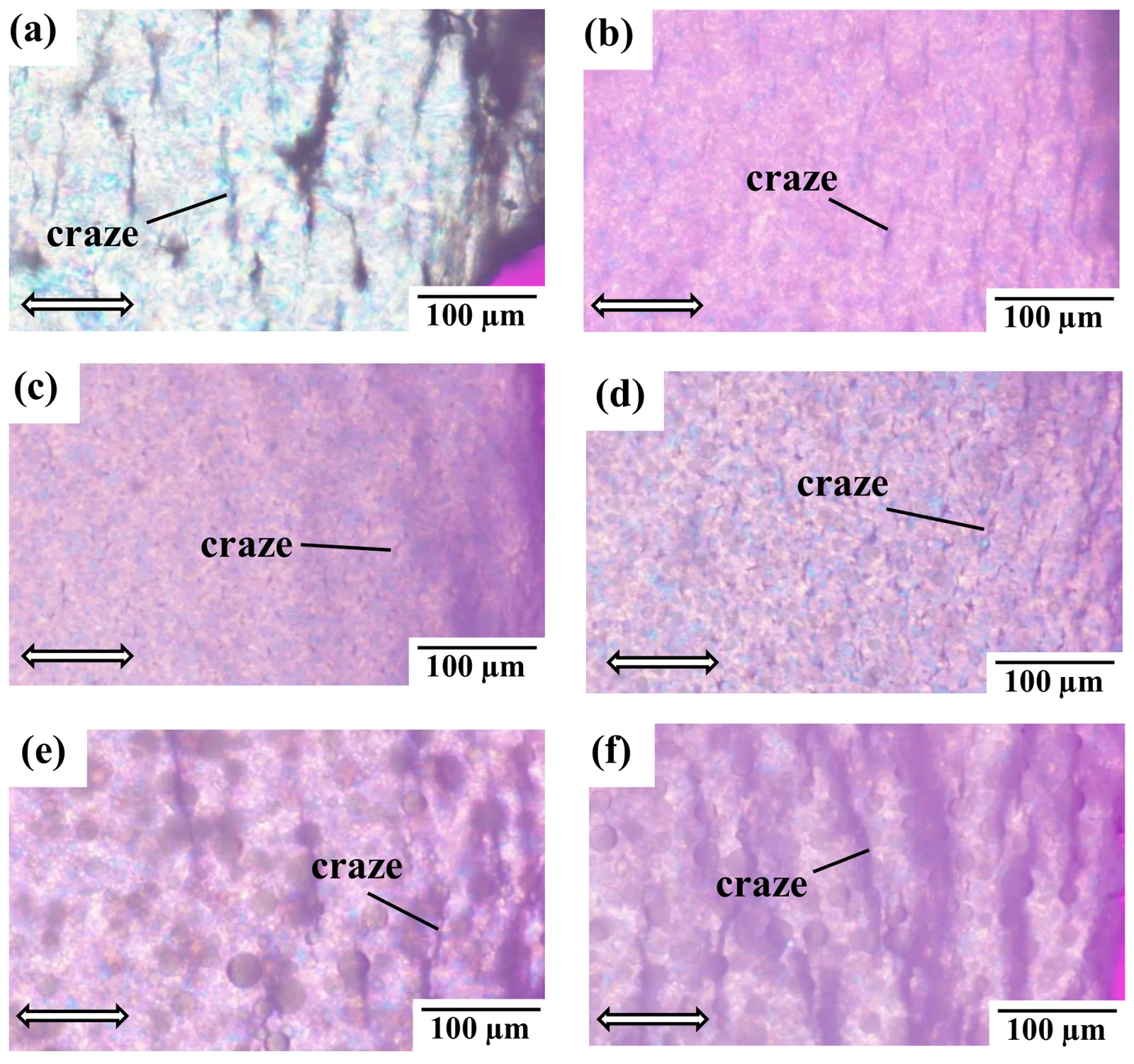
Polarized optical micrographs of PP/PBT blends with various compositions taken near the fracture surface after creep testing at 100 °C: (**a**) 100/0, (**b**) 100/1, (**c**) 100/3, (**d**) 100/5, (**e**) 100/10, and (**f**) 100/20. The arrow indicates the creep loading direction.

To elucidate the underlying microstructural damage under sustained loading, the deformed microstructures after creep testing were characterized. Figure 12 shows polarized optical micrographs taken near the fracture surfaces of the specimens after creep testing at 100 °C. Neat PP developed multiple distinct, elongated crazes aligned perpendicular to the tensile loading direction (Figure 12a). In contrast, no interfacial voids were detected in the low-PBT-content blends of 100/1 and 100/3 PP/PBT, and the crazes were substantially smaller and shorter than those in neat PP, suggesting that both craze initiation and microvoid formation were effectively suppressed throughout the prolonged creep process (Figure 12b,c). This structural stability is considered to reflect that the robust interfacial adhesion achieved by crystallization-induced mechanical interlocking derived from surface-induced nucleation effectively constrains the mobility of PP matrix chains at the interface. Consequently, the interface resists sustained stress and suppresses both the nucleation and growth of microvoids over extended durations, resulting in extended creep lifetime.

In the high-PBT-content blend, extensive, long crazes bridging coarse PBT domains perpendicular to the tensile direction were observed, originating directly from the surfaces of the coarse PBT domains (Figure 12e,f). Such long crazes bridging PBT domains were also observed in the 100/5 PP/PBT blend (Figure 12d) due to the coexistence of coarse PBT domains with a mean diameter of 10.7 μm alongside fine PBT domains (Table 1), suggesting that the failure transition occurs between the 100/3 and 100/5 blends. These observations indicate that premature interfacial debonding at the coarse PBT domains acted as primary stress concentration sites. Under continuous loading, the interface at the surfaces of coarse PBT domains failed to sustain the stress, generating an intense triaxial stress state in the intervening PP matrix (Figure 10b). This triaxial stress concentration restricted plastic flow and promoted rapid craze initiation and growth, thereby accelerating local deformation and leading to premature, weakest-link-dominated fracture triggered by the coarse PBT domains. The extensive opening and widening of these macro-crazes contributed to the large apparent fracture strain prior to final failure, as reflected in Figure 11. Although the creep protocol was limited to a single nominal tensile stress of 7.5 MPa, this specific stress level was selected to capture the competition between matrix plastic flow and interfacial debonding. Further multi-stress evaluations will be necessary to assess non-linear stress sensitivity and long-term failure transitions in future work.

## 4. Conclusions

Polymer blending represents a practical and economically viable strategy for upgrading post-consumer plastic waste via mechanical recycling. However, the thermodynamic immiscibility between constituent polymers typically causes weak interfacial adhesion, leading to premature failure under mechanical stress. We demonstrated that low-PBT-content blends (100/1, 100/3 PP/PBT) exhibited enhanced yield stress at room temperature, along with superior or comparable tensile ductility and extended creep resistance—characterized by both a reduced steady-state creep rate and an extended rupture lifetime—at an elevated temperature of 100 °C. These improvements are attributed to the robust interfacial adhesion established through crystallization-induced mechanical interlocking derived from the surface-induced nucleation of PP crystallites from small PBT domains. This robust interfacial adhesion was consistent with the high-temperature shift in the αc-relaxation reflecting enhanced interfacial constraint in DMA measurements. Microscopic observations and post-deformation thermal analyses clarified that matrix fibrillation preceded interfacial debonding during plastic deformation. This specific deformation sequence effectively dissipated local stress concentration, suppressing transverse craze propagation and enabling stable large-strain drawing despite the eventual occurrence of localized interfacial debonding. In contrast, while neat PP maintained ductile behavior at 100 °C, the high-PBT-content blend (100/20 PP/PBT) exhibited severe embrittlement and poor tensile performance. This brittle fracture is attributed to a weakest-link-dominated failure mechanism, in which premature interfacial debonding at the surfaces of coarse domains induces intense triaxial stress concentration prior to matrix fibrillation, inducing catastrophic macro-crazing and thereby accelerating creep failure. These findings provide vital mechanistic insights: the high-temperature fracture mode and mechanical durability of immiscible polymer blends are governed not merely by interfacial adhesion strength, but fundamentally by the competitive sequence between matrix fibrillation and interfacial debonding. Although this study utilized a model blend system consisting of virgin PP and PBT under laboratory conditions, controlling this deformation sequence through crystallization-induced interfacial engineering presents a novel, practical paradigm for designing tough, heat-resistant recycled polymer materials without requiring conventional chemical compatibilizers, which serve as future investigation into realistic post-consumer plastics containing degradation products and complex additives. Further investigations are required to assess how this crystallization-induced reinforcement translates into real end-of-life vehicle (ELV) recyclates that inevitably contain thermal-oxidative degradation products, residual additives, and particulate contaminants.

## Figures and Tables

**Table 1:** Quantitative morphological parameters of PBT domains.

Composition PP/PBT	Domain Size < 10 µm (Fine Domain)	Domain Size ≥ 10 µm (Coarse Domain)	Inter-Domain Ligament Distance
100/1	1.0 ± 0.3 (*n* = 32)	N.A.	N.A
100/3	2.8 ± 1.1 (*n* = 31)	N.A.	N.A
100/5	6.4 ± 1.7 (*n* = 35)	10.7 ± 1.0 (*n* = 7)	33.3 ± 24.9 (*n* = 8)
100/10	N.M.	15.4 ± 3.7 (*n* = 36)	19.1 ± 13.8 (*n* = 43)
100/20	N.M.	17.9 ± 5.7 (*n* = 39)	16.4 ± 9.2 (*n* = 57)

N.M.: not measured in the supplied dataset. N.A.: not applicable because no PBT domains ≥ 10 µm were observed.

**Table 2:** Tensile properties of PP/PBT blends measured at room temperature (*n* = 3).

Composition PP/PBT	Yield Stress (MPa)	Yield Strain (%)	Elongation at Break (%)
100/0	36.5 ± 1.0	6.3 ± 0.3	8.3 ± 1.5
100/1	37.5 ± 3.1	5.2 ± 0.6	7.2 ± 1.0
100/3	37.7 ± 1.1	5.6 ± 0.1	7.2 ± 0.9
100/5	34.8 ± 4.7	4.6 ± 0.4	5.3 ± 0.6
100/10	36.6 ± 2.2	4.3 ± 0.1	5.5 ± 0.4
100/20	(31.2 ± 0.9)	(2.9 ± 0.2)	4.1 ± 0.2

**Table 3:** Tensile properties of PP/PBT blends measured at 100 °C (*n* = 3).

Composition PP/PBT	Yield Stress (MPa)	Yield Strain (%)	Elongation at Break (%)
100/0	22.1 ± 0.8	12.8 ± 3.4	>600
100/1	22.6 ± 1.4	10.4 ± 0.0	>600
100/3	22.2 ± 1.5	10.9 ± 2.2	>600
100/5	22.4 ± 0.5	10.0 ± 1.7	308 ± 16
100/10	20.2 ± 1.0	8.1 ± 0.4	14.6 ± 3.9
100/20	16.6 ± 0.8	6.2 ± 0.7	8.8 ± 0.4

**Table 4:** Creep properties of PP/PBT blends measured at 100 °C under a constant tensile stress of 7.5 MPa (*n* = 3).

Composition PP/PBT	Steady State Creep Rate (s^−1^) ×10^−6^ (Mean ± SD)	Time to Failure (s) (Mean ± SD)
100/0	3.6 ± 1.9	3376 ± 810
100/1	2.0 ± 0.3	6491 ± 2311
100/3	8.3 ± 2.3	2607 ± 647
100/5	18 ± 10	743 ± 224
100/10	39 ± 17	682 ± 176
100/20	42 ± 10	867 ± 67

## Data Availability

The original contributions presented in this study are included in the article and Supplementary Materials. Further inquiries can be directed to the corresponding author.

## Supplementary Materials

The following supporting information can be downloaded at: https://www.mdpi.com/article/10.3390/polym18182282/s1, Figure S1: Time evolution of the integrated Hv (cross-polarized) light scattering intensity, *Q*_Hv_, of PP/PBT blends with various compositions during isothermal melt crystallization at 130 °C.; Figure S2: Schematic illustration of the determination of the αc-relaxation temperature from the onset of the *E*′ drop.; Figure S3: Polarized optical micrographs of PP/PBT blends with various compositions showing the regions near the fracture after tensile testing at 100 °C.
