# Supplementary material for: High-Temperature Tensile and Creep Behavior of Polypropylene/Poly(butylene terephthalate) Blends: Matrix Fibrillation and Interfacial Debonding
Source: Polymers (Basel). 2026 Sep 18;18(18):2282. doi: 10.3390/polym18182282 (PMC13611715; doi:10.3390/polym18182282)
Supplement: Supplementary file 1 [file polymers-18-02282-s001.zip › polymers-4556029-supplementary.pdf]

Supplementary Materials

High-Temperature Tensile and Creep Behavior of Polypropylene/Poly(butylene terephthalate) Blends: Matrix Fibrillation and Interfacial Debonding

Mio Kudo[1,2], Mai Ishikawa[3], Hirotaka Horiguchi[1], Shinya Goto[1], Hiromu Saito[2] *

[1] Materials Engineering R & D Division, DENSO CORPORATION, Kariya-shi 448-8661, Aichi, Japan

[2] Department of Applied Chemistry, Tokyo University of Agriculture and Technology, Koganei-shi 184-8588, Tokyo, Japan

[3] Department of Industrial Technology and Innovation, Tokyo University of Agriculture and Technology, Koganei-shi 184-8588, Tokyo, Japan

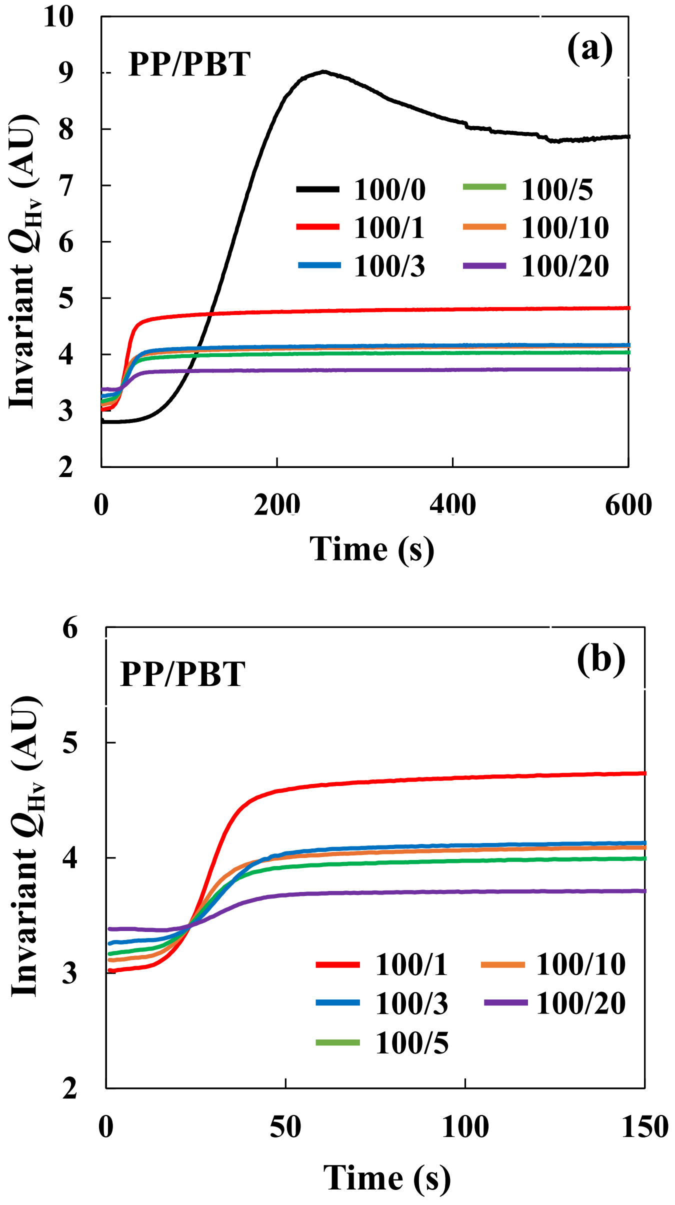

Figure S1. Time evolution of the integrated Hv (cross-polarized) light scattering intensity, Q_{Hv}, of PP/PBT blends with various compositions during isothermal melt crystallization at 130 °C: (a) long-period region, (b) short-period region. Details of the experimental method are described in Reference [43].

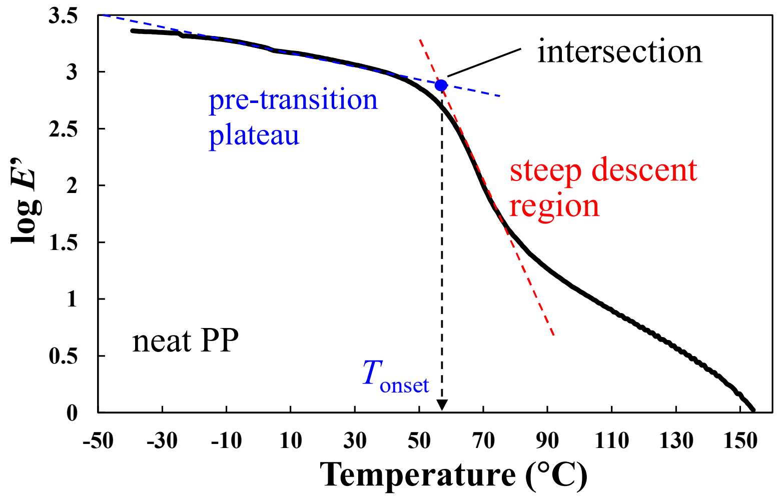

Figure S2. Schematic illustration of the determination of the αc-relaxation temperature from the onset of the *E*′ drop. The onset temperature was quantitatively identified from the intersection of two linear tangents: one along the pre-transition plateau and the other along the steep descent region of the *E*′ curve.

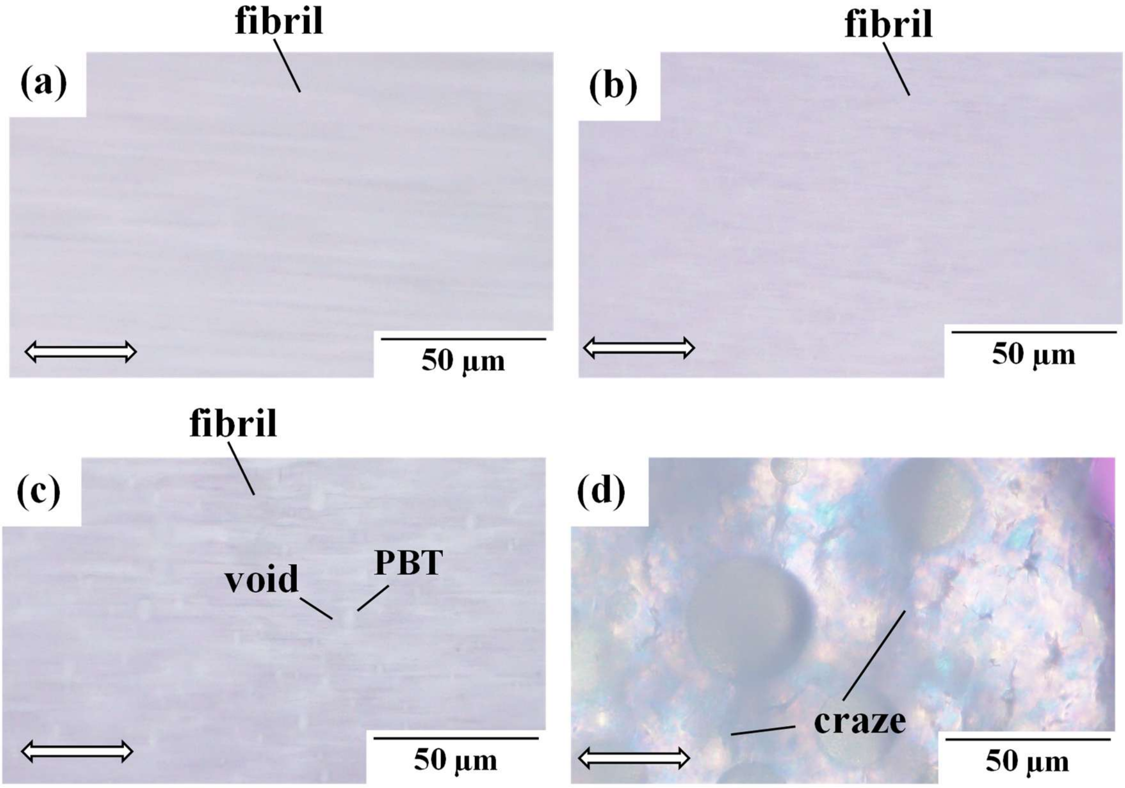

Figure S3. Polarized optical micrographs of PP/PBT blends with various compositions showing the regions near the fracture after tensile testing at 100 °C: (a) 100/0 (neat PP), (b) 100/1, (c) 100/3, (d) 100/20. The arrow indicates the drawing direction.
